# Discussions With End Users to Inform the Vision for a Shared Care Record in Ontario: Qualitative Interview Study

**DOI:** 10.2196/51231

**Published:** 2024-02-27

**Authors:** Marta Chmielewski, Matthew J Meyer

**Affiliations:** 1 Public Health Program Schulich School of Medicine & Dentistry Western University London, ON Canada; 2 Middlesex London Ontario Health Team London, ON Canada; 3 Office of Health System Transformation London Health Sciences Centre London, ON Canada; 4 Department of Epidemiology and Biostatistics and Interfaculty Program in Public Health at the Schulich School of Medicine and Dentistry, and the Ivey Business School Western University London, ON Canada

**Keywords:** population health management, shared care record, health information exchange

## Abstract

**Background:**

Improving the health outcomes of populations of individuals through population health management requires the use of electronic health records that can exchange real-time digital information using an accurate and complete shared care record that is accessible to health care providers, services, and patients.

**Objective:**

The aims of this study were to understand end users’ (health care providers) experiences, attitudes, and insights using current electronic health records; their expectations of what is required to establish a shared care record; and how they anticipate adapting to the use of a shared care record in daily practice. This work is the result of a quality improvement initiative deemed not to require ethics approval according to the Western research ethics board checklist.

**Methods:**

Clinicians were contacted using voluntary response sampling and interviewed via Zoom (Zoom Video Communications) between June 2022 and July 2022. The participants were from various health care sectors and at various stages of career development.

**Results:**

Overall, adaptation to the use of a shared care record was viewed positively by health care providers, highlighting the benefits of a centralized, shared, and accessible location for real-time data, promoting patient continuity of care. The main concerns included the privacy, confidentiality, and security of the record along with patients’ ability to interpret their own medical information found in a patient portal. The resources requested by end users included multifaceted ongoing training on the use of a shared care record.

**Conclusions:**

This study provides practical findings that will help emphasize factors that facilitate clinicians’ practical use and process of adaptation to the use of a shared care record.

## Introduction

### Population Health Management

Defining population health management (PHM) requires taking a step back and thinking of the bigger picture. The Population Health Alliance PHM framework explains that “a population health management program strives to address health needs at all points along the continuum of health and wellbeing through the participation of, engagement with and targeted interventions for the population” [1]. Their definition specifies that the goal of PHM is to uphold or improve the “physical and psychosocial wellbeing of individuals through cost-effective and tailored health solutions.” Breaking it down, PHM involves the use of data to proactively manage the health and well-being of an identified population of individuals while considering the diversities within that population along with their social determinants of health [2]. PHM is a constantly progressing concept that is increasing in popularity worldwide. For example, in Ontario, PHM has been characterized as a fundamental element in Ontario’s health system transformation. In the Netherlands, several PHM initiatives are working to tackle the health-related social needs of residents by building partnerships among medical care, public health, social services, and community-based organizations [3]. Managing populations of patients based on their diagnosis while maintaining their health and keeping them out of dangerous circumstances has recently become popular as it affords the ability to deliver high-quality and efficient care that is satisfying to everyone involved [4]. Examples of leaders in PHM and integrated health care delivery include those in Denmark, Spain, and the United States, such as Geisinger, Memorial Hermann, the Department of Veterans Affairs, and Kaiser Permanente. Another important mention is Epic, a widely used software company among hospitals that allows for the exchange of medical records across organizations in the United States and beyond. They are all highly regarded for high-quality and efficient health care through integrated care delivery processes [5].

PHM can be viewed as improving the health outcomes of a population using appropriately coordinated care and proper patient engagement, which is sustained through adequate economic and care models [6]. The question then becomes how to best support the entire population clinically and financially. According to the *Health IT Playbook* [7], examples of PHM services involve efforts to proactively help people improve their health, guarantee they obtain preventive screenings, and help them effectively manage their chronic conditions. A vital feature of this approach to care delivery is that the population whose health is being managed is a complete group of people, not only those who are pursuing health care. This population can be defined in ways such as all the employees of an employer, members of a health insurance plan, or residents of a community, but the key feature in PHM is that the health of all members of this population is considered [7].

Another crucial aspect of PHM is coordinating a diverse and progressive group of stakeholders who work together to provide programs, services, and tools for interoperable care for patients in various health care settings [8]. This is also where the integration of services occurs, such as financing and delivery of health care working together [4]. According to Jones and Smith [4], an entirely integrated care system is defined as both horizontally and vertically integrated. Vertical integration combines provider and care delivery, financing, and support services such as IT. Horizontal integration combines provider services, home health services, hospitalization (tertiary and secondary), and ambulatory care, entailing continuous and seamless care [4].

### Health Data

The growing burden of chronic diseases challenges health care system sustainability in countries worldwide. Working toward coordinating care to prevent unnecessary hospitalizations is a crucial solution to limiting increasing health care costs. According to Burnel [9], to reach this goal, clinicians and professionals must be able to exchange information using electronic health records (EHRs). An EHR is a real-time digital form of a patient’s health care record, allowing information to be available to providers authorized to access it across different health care organizations instantly and safely [10]. Beyond providing a patient’s collected medical data, an EHR offers a comprehensive view of a patient’s care. An EHR contains information from all providers involved in a patient’s care concerning admission documents, diagnostics, ongoing assessments, and health care plans and can be shared with other health care providers; caregivers; patients; and organizations, including laboratories, medical imaging facilities, specialists, pharmacies, and clinics [10]. EHRs make it possible to reduce medical errors, increase health care provider communication, and improve care coordination [11]. The broad implementation of EHR systems in primary care has permitted the compilation of enormous amounts of clinical data that have the potential for secondary use, such as improving clinical programs, system management, and population health research [12].

### Shared Care Record

With EHRs in mind, the concept of the *shared care record* is introduced. A shared care record is an enabler that helps allow PHM to be possible. According to the Patient, Family, and Caregiver Declaration of Values for Ontario [13], to enable integrated care, each resident in Ontario ought to have access to their health-related information record, which is “accurate, complete, available and accessible across the provincial health system at [their] request.” The record should be accessible to health care team members and patients as required and in a manner that encourages appropriate care and positive experiences. A complete and accurate shared care record includes up-to-date information about the person and their demographic information, the administrative services they use, their medical or clinical information, and additional health-related information involving the social determinants of health.

Shared care records permit all primary and secondary care providers to view and use a single dependable source of documentation that is up to date and provides accurate clinical information in real time about a patient [14]. It is about giving everyone access to the information they need but does not require everyone to be on one common information system. Patient records from a variety of care providers and sources can be linked through a health information exchange (HIE) system. The vision of the shared care record would give providers, in the home database system they work in daily, access to information captured about their patients from other care providers and other systems. For example, a provider (or patient) can view medications prescribed by provider A alongside those prescribed by provider B in the same place even if providers A and B use different EHR technologies. This information could come from their primary care records, home and community care records, community mental health and addiction records, or hospital systems. Systems worldwide are using this approach to link information on allergies, laboratory test results, procedures, appointments, and much more. The HIE simply enables information exchange between systems, for example, between hospitals and primary care [15]. Moving this information between the systems aims to help the care team locate and use the correct information to provide safe, efficient, and equitable patient-centered care. This means that a patient only needs to describe their health care history once instead of sharing it multiple times at each health care encounter. If done properly, information from this system can also provide information to public health teams to understand the health and health needs of the population [15].

### The Need to Reform Service Integration in Ontario

The current health care system in Ontario is experiencing increasing strain from the aging demographic, overloaded hospitals and emergency departments, and a significant increase in chronic diseases, putting our care delivery model at risk [16,17]. Completely changing the Ontario model of health care delivery is not feasible; therefore, we must work with the existing structures. One example is health IT systems that can be better connected to improve workflows; centralize health data; and deliver information to health care providers, patients, and families where and when they need it. Ontario is not alone; fragmented care exists among health care systems worldwide involving a lack of communication between primary care physicians, other health care providers, specialists, patients, and families, leading to negative impacts on patients and gaps in continuity of care. Many systems have realized the benefits of interoperability, “defined as the ability of different health information systems to cooperatively access, integrate and exchange data to advance effective delivery of health care” [16]. Several obstacles must be kept in mind when it comes to the electronic exchange of health information, such as technical, financial, legal, and privacy barriers that can impede the implementation of interoperability. Nonetheless, as health care providers request continuous integration of information and patients stress the need for access to health data, health care organizations will be forced to share information appropriately. This may require funding for information management technology such as EHRs and IT to enable care across the continuum [4,17].

The concepts of integrated care, digital health, interoperability software, and centralized health data, exemplified by the shared care record, are crucial to exposing the benefits of a restructured and better coordinated health care system. Collectively working toward a shared care record can help reduce medical errors, health care costs, and redundant and unproductive work while improving communication among health care providers, quality of patient care, and seamless transitions of patients across health care providers and settings to create a resourceful system [17,18].

### Aim of the Study

This qualitative study used semistructured interviews to improve the understanding of end users’ (health care providers) perspectives and insights regarding how they anticipate adapting to the use of a shared care record. Information gathered from the interviews will support the development of use case storyboards to inform various stakeholders across Ontario of considerations for developing a shared care record across the province. Talking with end users will help understand what a range of clinicians from different specialties believe is required to establish a shared care record and how they will adapt to its use over time.

## Methods

### Setting

Middlesex County is in Ontario’s Southwestern region, covering a geographical area of 2800 km^2^ and home to >450,000 people. This region consists of a mix of urban and rural residents. London is the largest metropolitan area within Middlesex County and is home to >450,000 residents. The region also surrounds 3 sovereign First Nations: the Chippewas of the Thames, Oneida Nation of the Thames, and Munsee-Delaware Nation. For several years, legislators in Canada’s most populated province, Ontario, have endeavored to change the local health care system to create a more coordinated and financially united system [19]. This initiative resulted in the Government of Ontario Ministry of Health formation of 54 approved Ontario Health Teams (OHTs) within specific geographic areas across the province. OHTs modify how health care is financed and delivered and concentrate on collaborative partnerships in which providers and organizations such as primary care, mental health services, hospitals, and home and community care work as one synchronized team [20,21]. The Middlesex London OHT is specifically responsible for supporting the health of the population residing in the Southwestern Ontario region [22]. Using OHTs, the provincial government is assembling sustainable systems that will respond to local populations’ short- and long-term needs, support local services, and enable straightforward system navigation and transition among providers [19,23]. Another critical player providing guidance and regulation is Ontario Health, a government-formed agency working to coordinate and connect the province’s health care system [24]. This new visualization of Ontario’s health care system is aligned with the Quadruple Aim, a framework internationally understood to design and provide a system that improves patient and caregiver experiences, patient and population health outcomes, and provider experiences while reducing total costs [23].

### Participants and Recruitment

Using voluntary response sampling, clinicians were contacted via email based on preexisting professional relationships. A total of 14 health care providers were interviewed, comprising those who volunteered or agreed to participate upon request. These health care providers hold positions in various care sectors, including nursing, community care, primary care, emergency medicine, dietetics, practice specialties, occupational therapy, and physiotherapy. The list of health care provider interviewees who agreed to participate in the data collection, organized by occupation, is shown in Table 1. The providers also ranged widely across stages of career development and duration, from new graduates to experienced employees.

**Table 1:** Interviewees.

Professional title	Care setting	Professionals (N=14), n (%)
Registered nurse	Inpatient acute care	3 (21)
Registered nurse	Cardiac outpatient clinic	1 (7)
Registered nurse	NSWOC^a^	1 (7)
Clinical nurse specialist	Chronic diseases and clinical informatics	1 (7)
Clinical dietician	Bariatric outpatient clinic	1 (7)
Physician	Emergency medicine	1 (7)
Physician	Primary care practitioner	1 (7)
Registered physiotherapist	Outpatient clinic and inpatient acute care	1 (7)
Registered practical nurse	Home and community care	1 (7)
Clinical practice specialist	Occupational therapy	1 (7)
Clinical practice specialist	Palliative care and oncology	1 (7)
Occupational therapist	Home and community care	1 (7)

^a^NSWOC: Nurse Specialized in Wound, Ostomy and Continence.

### Data Collection

Participants in the study were first introduced to the concept of a shared care record verbally and through a video demonstrating its functionality. All participant questions about a shared care record were answered before the interviews. A semistructured question guide ensured that each interview covered essential topics and allowed participants to disclose issues and stories as they saw relevant. The use of a prepared guide also worked to decrease interviewer bias by decreasing interviewer involvement. Confidentiality and anonymity were established at the beginning of the interviews. Verbal consent was obtained from each participant to potentially use quotes from the discussions in future publications or presentation materials that result from the initiative. The semistructured interviews averaged 15 (SD 2.56) minutes and were web-based via Zoom (Zoom Video Communications) between June 2022 and July 2022.

The interview format was chosen, as opposed to focus groups, as it allowed for direct, individual engagement with each end user. Stokes and Bergin [25] discussed the opportunity for the interviewee to truly analyze their motivations for a particular action while being given a feeling of empowerment because of the anonymity in the individual interview setting without the pressures of a group setting that may lead to a consensus view. The interviews were designed to elicit the health care providers’ understanding of and experiences with the EHRs they currently use along with their attitudes, beliefs, and expectations regarding the future use of a shared care record in their daily practice. The sequence of interview questions used and additional instructions to guide the interview are shown in Table 2. The interviews were audio recorded with permission from the participants, transcribed using web-based software, checked for accuracy, and then analyzed to develop a report. The results present the participants’ initial reactions to the concept of a shared care record and then transition to their interpretation and reflections on the use of and adaptation to a shared care record.

**Table 2:** Interview guide.

Interview portion	To do	Additional notes
Warm-up	Introduce and explain the purpose of the interview. Obtain consent to use quotes from the interview and to record the interview.	Introduce the idea of the shared care record and how it works.
Interview questions	Consider your electronic health record today (name the record), what additional health or social information regarding your patient would you like to have access to in this new shared care record or would make a difference for you, when providing care for that patient? Prompt: tell interviewee more about what a shared care record could offer them. So, for you, tell us how this information would impact or change the care you provide (or can) to your patients? Prompt: what difference would it make if you had access to all of your patient’s information (the type of information you just listed) on the shared care record? Prompt: would you look at it more or make use of that information? This would be a change and change is never easy, but what do you think you and your colleagues would need to do to adapt to using a shared care record in your day-to-day practice? Prompt: what would help your colleagues benefit from this change? Prompt: what support or resources would help you with the introduction to and adaptation to this system? Do you have any concerns with the concept of a shared care record? Prompt: Overall, what factors or conditions challenge or serve as barriers to your personal use of a shared care record? How might this change that? In closing, do you have any concluding thoughts or comments related to the shared care record that you would like to convey?	Build each question off the previous one and rearrange the order as needed according to the flow of the conversation. Use the prompts to further stimulate conversation.
Closing	Thank the interviewee for their time. Let them know not to hesitate to reach out if they have anything else they would like to discuss.	Ask interviewee whether they give permission to be contacted in the future.

### Data Analysis

The interviewer first read and interpreted the individual transcripts to become well acquainted with the data collected. After developing the initial semantic codes based on the data, the interviewer grouped the codes into categories and themes and then reviewed, named, and discovered various connections between the themes to write the analysis. The themes were modified using an iterative process, adjusted, and grouped, with categories and subcategories added as they arose from the data analysis. The interviewer used a qualitative interpretative approach, the framework method, to analyze the data by joining thematic analysis with comparison so that the data were surveyed for known literature themes and emerging themes. The interview findings are presented in the *Results* section of this paper; quotes are included from the interview transcripts to illustrate the generated themes. End users are identified by their health professions in each quote.

### Ethical Considerations

This publication is the result of a quality improvement initiative deemed not to require ethics approval according to the Western research ethics board checklist.

## Results

### Participants’ Reflections on the Shared Care Record

#### Theme 1: Opportunities for Using a Shared Care Record

##### Lack of Communication Affecting Care Delivery

A lack of adequate communication among health care providers, services, and health care facilities across the care system was a common response among participants. Discussions with end users highlighted frustrations across the care continuum, such as entering care encounters with inadequate or lacking information, more difficult care management and planning, and delays in access to information causing delays in care. Registered nurse 4 stated the following:

Why are we doing the same assessments over and over again? Patients are forced to repeat tests because the results are not passed between the health care providers, which in the end only delays their [the patients’] treatment.

The introduction of a shared care record could drastically improve communication among health care providers, potentially decreasing the current workload, increasing confidence in decisions, and affecting patient safety and continuity of care:

...with a client that I’m seeing, he’s a cancer patient, so he’ll go to London, and then he’ll go to Stratford for example. The two hospitals they don’t communicate very well...the communication between the two kind of gets lost in between...it’s all of these extra steps between myself and this patients’ daughter. We are trying to figure out when was his last treatment? And was the medication provided? What was that medication? And how long was he supposed to take that? So, you have Stratford asking this and trying to get through to London to get those questions answered, the whole process becomes very difficult.

Registered practical nurse

End-user discussions touched on the impact of poor communication on patient satisfaction, trust, and their subsequent health care journeys. Occupational therapist 1 described how enhanced communication between providers could affect the patient care experience:

From a patient perspective, it might be one less time they have to answer the same questions. Not being asked the same questions all over again seems trivial, but if you’re the patient who’s had to answer the same question twenty times you think people aren’t listening to you...we could just kind of summarize what we know, which I think also makes the patients feel like we’re all a team speaking with each other. So, we’re communicating what we know about the patient, as opposed to having to ask them the same information over and over and over again.

The retrieval of information to provide proper care was deemed exceptionally crucial among end users working in community settings, where some may not have any connections to EHRs:

I mean our nurses are walking in really with very minimal information. Again, relying on the family a lot of the time to tell us, even as far as medications that they’re on, you know, we’re going through all of their bottles and discharge lists, and lists they pull out of their wallet, and trying to reconcile it. So, it’s really pieces of information.

Clinical practice specialist

##### Real-Time Information

End users collectively admired that one of the most critical advantages of the record would be the access to real-time information as it changes and becomes updated. A clinical nurse specialist described information access as “very much a game changer for clinicians,” with other participants agreeing, describing it as “taking the legwork*”* out of obtaining essential patient information. The shared care record real-time information feature “would not only help the patients but also the healthcare team be up to date, and they wouldn’t have to take so long to find the information they need” (registered nurse 3). Considering their daily practice allowed the end users to visualize clinical patient data being stored in one central location where the information could be accessed, analyzed, uploaded, and used, with one process going to everybody involved in the patient’s care:

I’m really intrigued by receiving information in real time, I find that especially in the community if there has been a medication that’s been added, I don’t always receive that information, unless I’m at the client’s home and I see the new bottle because they don’t typically tell me if they are on a new medication, and the doctors just prescribe it, they definitely don’t inform us. That would be information would be very helpful for me when trying to figure out why they’re having a change in their health status, or maybe a cognitive change or something along those lines.

Registered practical nurse

#### Theme 2: Perceived Benefits of Using a Shared Care Record

##### Effective Use of Time

Access to clinical information through the shared care record was described as promoting the effective use of time and resources. End users felt that the record would provide “an accurate picture of what’s going on,” and it would be “a lot less doubling and tripling of assessments.” Registered nurse 1 recalled a common scenario occurring in their inpatient hospital unit:

...on my floor patients come up with a bag of medications or just a list of medication names and dosages that they have scribbled onto a piece of paper...the time we are spending on doing something very basic like manually inputting medication information that should be available through the pharmacy or from a physicians list would save so much time and then you would be able to spend more time doing a proper assessment on the patient, providing care or starting a treatment.

Within the complex and fast-paced acute care setting in which several of the interviewed end users worked, the ability to save time was the most significant determinant of efficiency. Effective care must be provided with often limited resources and high workloads. Occupational therapist 1 discussed the vision of the shared care record as a benefit to their work:

...we’re always looking in acute care for efficiencies. The length of stay is already very short, and the more information that we have access to when we are doing our initial triage or chart review, the earlier we can start to at least reflect on what the likely plan is.

##### Informed Circle of Care That Promotes Continuity of Care

The vision of the shared care record would allow the patients’ circle of care, everyone involved in the patients’ care, to be well informed, leading to better care, time savings, and less frustration. According to registered nurse 1, “it would promote continuity of care and keep everybody in the loop and informed, which is so important in healthcare in general.” End users discussed the benefit of being able to collaborate with other clinicians and share more information in general:

...even when our clients go into hospital, they [the hospital occupational therapists] have no idea what we have been working on at home. And then if they go to discharge the client, sometimes they’ll put in new OT and PT services without realizing there’s already different things in place. There’s just poor communication, so I think this idea would make a huge difference.

Occupational therapist 2

The consensus among the participants was that this vision becoming reality would change the way they practice, offering the ability to connect with everyone that the patients are in contact with:

...everything is just in silos right now, and I think anyone that’s had any contact with the healthcare system knows that. I think it’s very prominent in community...it is not practical how it is right now, so I think any move in that direction [access to a shared care record], would help immensely.

Registered nurse 4

With regard to the patient-provider relationship, providers indicated that patients in the hospital setting can feel vulnerable and left out of conversations and might not know what is going on:

If we could retrieve those records...it would help the patients feel comfortable, they would be able to ask more questions during their stay and obtain answers from us as providers.

No one wants to be in the hospital as it is, so when they [the patient] come in, and they notice that their cardiologist has no idea that so and so [other healthcare providers] prescribed a certain medication, patients tend to become annoyed, and rightfully so...having that information prior to their visit would make their visit a lot easier, faster, and more efficient for them and for everyone involved.

Registered nurse 5

##### Overview of Patient Health Status

The increased amount of health and health-related information accessible to health care providers would help them understand their patients’ medical requirements. A physiotherapist discussed how treatment of his patients would be enhanced “by helping me know and understand their timeline for recovery.” The record would “help understand other areas they [the patient] need help with because patients forget things and don’t always understand what other healthcare providers tell them when it comes to their injury.” Participants noted a lack of patient awareness regarding what providers are involved in their care, medication management, or even their diagnoses. Physicians discussed situations in which, unless their patients informed them, they were unaware of new allergies or changes to medication dosing made by other physicians. Registered nurse 2 discussed that, when noticing a new irregular sign or symptom, the shared care record would allow for “a quick reference, if that’s something they [the patient] have at baseline or if you need to look into it further, and just kind of base your actions on that information”:

...especially going into people’s homes, it would just make me more aware of things. People won’t always tell you the truth about things or they’ll leave out things they don’t feel are important but are impacting how they are managing at home...If you knew the information, walking into it, you kind of have a more holistic picture before moving forwards with them.

Occupational therapist 2

### Adaptation

#### Theme 3: Factors That Promote Use of the Record

##### Positive Outlook on Adaptation

Overall, the participants revealed an incredibly positive outlook when considering their own and their colleagues’ adjustment to the record. Participants made comments such as “it wouldn’t be a challenge for me and my colleague’s personally” (registered nurse 3) and “I don’t think there would be a large change-related level of concern or anxiety” (occupational therapist 1). End users acknowledged that technology is on the rise, with many individuals of all ages using digital solutions in their daily lives:

...even just logging in and seeing their bloodwork online, people are more comfortable doing that...even if they’re older or they haven’t done that they’re comfortable reaching out to their neighbor or their child to help them with that...I think it would be easier than you know even 5 years ago.

Registered nurse 4

##### Health Care Provider Requests for Health Information Access

When end users were asked what other patient health or social information they would like access to when providing patient care, most made remarks regarding the difficulty of obtaining access to clinical data documented beyond the organization they worked within. The most common request among end users was access to a verified list of medications. Often, providers must rely on medication bottles, discharge lists, and family members to reconcile patient prescriptions:

...patients might know the name of the drug, or what the pill looks like, but they have no idea what it does, or why one of their physicians ordered it. For example, when they come into the clinic telling us that their nephrologist ordered something to bring their blood pressure down because their kidneys are failing, their cardiologist might just be finding out about the medication and realizing the medication could be affecting their heart. So being able to see what was ordered and when it was ordered would be such a big help.

Registered nurse 5

The lack of integrated health IT was found to cause duplication of efforts and lack of comparison across documentation from the hospital versus the community or across organizations or different regions:

I cannot see pictures of x-rays that they [patients] get in the community. So, people get sent in with a break and I need a picture to see if I need to push on it, to put a cast on it and get it in the right place. We often repeat x-rays that probably wouldn’t need repeating if we could just see the original picture.

Physician 1

Interoperability between organizations can reduce redundant tests, save time and costs, and result in better continuity of care. Participants regarded patient diagnostic imaging as the information they would like to access, including the actual pictures and not just the reports of x-rays, ultrasounds, computed tomography scans, echocardiograms, and other imaging modalities. Furthermore, patient exposure to radiation or contrast dye would decrease without the need to repeat tests and scans:

...it would help me if I had access to imaging without having to rely on what the patient tells me or what they can even remember. Sometimes patients don’t even know what type of imaging they had done or even what that imaging was for.

Physiotherapist

Participants listed patient medical-related appointments as the information they wanted to see on the record. Many patients have complicated cases and multiple teams following them in the community, which can be overwhelming to manage independently. Family physician records or membership in a family health team was another common request, along with up-to-date access to all referrals sent out and specialist information:

Knowing what doctor the patient was referred to...a lot of time people will come in and will say “well my doctor sent me to a cardiologist, but I haven’t heard anything in three months” So then I’m like “well I’ll send you to a cardiologist as well.” Am I sending them to a different cardiologist? I have no idea.

Physician 1

Access to patient social history information was highly requested, including living arrangements, home care reports, and community support or professional services that patients were using as this information significantly affects patient care planning and discharge planning. According to physician 2, “it would be nice if the patient could update things like occupation, substances, family members, consent to family members”:

In many cases, we don’t have a true understanding, at least initially on chart review, without speaking with our team about the exact specifics of what type of services or equipment or programs they are [the patient] currently involved in, in the community. A true understanding of that social and community history allows us to initially strike off maybe some options that we may not have at our disposal, or start to plan out some of the gaps that we anticipate based on what we know that they already have.

Occupational therapist 1

End users discussed situations requiring access to patients’ medical histories and complete health records. Requests for clinical data access included a complete list of diagnoses and when the patient was assessed for them; laboratory test results; previous rehabilitation journeys; surgeries; and conversations that had taken place, which could indicate the patient’s understanding of their illness or where they are at:

Almost every single clinician that I have spoken to would say, we wish we had more, or the information is just a very brief summary...especially with acute care, the length of stay is so short, we’re trying to piece together as much as we can...so the more the more understanding of the patient’s history and journey through the healthcare system, the more efficient we can be as occupational therapists.

Occupational therapist 1

##### Ongoing Training

The most common suggestion among end users was the provision of proper education and training on the new system. As one of the primary objectives of an EHR is to improve collaboration among health care providers, it only makes sense that they are offered the chance to provide feedback on the system they use daily:

From a training perspective, it’s nice to first of all be part of the process of building the system, or having some input on that system, which helps with the engagement and integration when we’re actually putting the rubber to the road...it definitely would help with the connection to the implementation.

Clinical nurse specialist

Most participants suggested that they would benefit from getting to know the new system through proper training on the layout of the information and how to find the information that they could use. End users visualized the benefits they could obtain from an introduction to and familiarization with the system before it becomes implemented in practice:

...obviously, there needs to be training, and along with that comes the resources. Not only do the training but pay for them [the end users] to attend, which is always an issue. And then I think even support along the way, for example IT support, do we need to build that internally in our IT department...up front, it’s just really the education and making sure it is ongoing...in healthcare in general, there is a lot of turnover, so how do we sustain the education moving forward.

Clinical practice specialist

Many participants discussed “multi-pronged approaches” as the most effective method for introducing and adapting to the record. Resources mentioned by the participants included a chat or live support option for immediate questions, videos on how to use the system, a toolkit or tip sheet developed by the system creators, and in-person and web-based computer sessions. Occupational therapist 2 described the introduction to their current EHR system as they recalled:

Clinicians felt more comfortable using the system if they had some test patients to go through trial cases of what a daily patient intervention might look like prior to the go live.

Several participants mentioned the idea of “super users”:

...our nurse colleagues on the floor, who had additional training and were more familiar with the record so that we could reach out to them if we needed help or if we had questions.

Registered nurse 1

These super users would function to support their colleagues in the transition while helping others learn to use the record to the fullest extent.

Another resource identified was the use of clinical educators who already work to support staff with clinical updates to rules, procedures, policies, and methods of accessing information.

##### Record Accessibility

Several questions from end users concerned how to physically access the clinical data on record and the timeliness of finding information. Questions included the following: “where do we need to click?” “Under what icon?” “How do I add things to the shared care record?” “Do I have to do it manually?” “Does it just happen automatically?”:

The biggest adaptation would be how to access the information, like opening the charting system will look different, so getting used to the new layout and the new system and knowing where to find things.

Physiotherapist

A key finding among participants included statements regarding the user interface or usability of the shared care record. Participants used terms such as “seamless,” “simple,” “accessed quickly,” “user friendly,” and “easy to follow” to describe how they envisioned the software to function:

But we need to really limit where they’re [frontline staff] finding their information, if they need to upload, that they are not having to do it to all of the people we need to report to. There are so many layers and rules, and we just need to make it as simple as possible.

Clinical practice specialist

Participants considered the least amount of clicking and integration with their current systems to obtain data or add to their assessments as crucial features of the record. Information being uploaded automatically was considered foundational, with physician 1 commenting the following:

If we have to do an additional step at the end to get it uploaded, you’re going to get way less uptake...as long as in the back end of things, my EHR links it all up.

Regarding user-friendliness, the physicians explained that they would not appreciate retyping a password to access the charting or repeated verification of the designation upon entering the system:

I don’t want to have to log into something else, I’m already logging into so many things every day...and so there’s that information that sits somewhere but that it gets pushed to all the different places and then shared between the different places.

Physician 2

#### Theme 4: System-Dependent Considerations and Concerns

##### Clinical Data Consistency, Accuracy, and Organization

Although participants recognized numerous potential benefits, they also discussed fundamental considerations of functional practicality, such as the consistency and accuracy of data across the record. To present clinical data across different EHR systems uniformly, health care providers must be consistent with the documentation methods and upload the documentation to the record:

I would be concerned about it being unorganized or messy, um if everyone has different styles and systems of taking notes or recording, maybe it would be difficult to find one particular piece of information that you are looking for.

Physiotherapist

Understanding patient rostering or enrollment was discussed as a critical element of the record, understanding who is involved in a patient’s care, and participating providers can change or adjust that if needed:

I changed my practice maybe three years ago, but I am still on some people’s charts at the hospital. I have requested to have my name taken off, but unless the patient calls and changes that, nothing can happen. And so that becomes a privacy issue, I keep getting files for people who I am not actually taking care of.

Physician 2

Beyond consistency, participants identified the importance of double-checking the information obtained from the record with the patients themselves. Updates may not be revised, data could be deleted, and mistakes can still occur:

If you have a medication record from two years ago, you would still have to do your due diligence to make sure the information you are using is accurate.

Registered nurse 1

An auditing system of the record was suggested that could review charting to help ensure that health care providers input the required information to maximize the utility and reliability of the clinical data. According to registered nurse 5, “that way people have to take responsibility for what they’re changing or what they’re contributing towards this shared documentation.”

##### Change in Workflows

Uncertainty regarding daily practice workflows came up as a barrier to overcome when participants discussed adaptation to the use of the record. Visualizing the details of the change to their current EHR system interface was difficult for certain participants:

It’s hard to know what the change management strategy would be.

Clinical practice specialist

My hope is that there would be very little that we would actually have to change...everything else I would expect to be kind of behind the scenes where I do my normal process, that it would just sort of happen in the background.

Physician 2

Discussions held with professionals in the community setting revealed that there would be an adjustment to their current workflow, with the additional time spent reviewing history, reports, and other data accessible in the record before going in to see the patients:

Right now...I really only check [name of EHR] to look at when appointments are confirmed, phone numbers, names, and then I find out a lot more information from the patient once I get there [to their place of residence]...for home care, that’s just kind of how it’s been. If I am seeing four or five people every day and driving between these destinations, it would take time maybe at the beginning before getting used to it as part of the routine.

Occupational therapist 2

Upon introducing the idea for the record, a statement from the Ontario Patient, Family, and Caregiver Declaration of Values [10] was presented to participants explaining the vision for transparency in patient access to their health records. Wondering how patients would interpret seeing physicians no longer in the patient’s circle of care re-entering their medical information and whether this would be concerning, physician 1 stated the following:

...Most of us [physicians] require understanding of, did the treatment I gave actually have a good effect? And what did the follow up doctor think? So, we will access records a few weeks later to see what happened so that we can learn...that’s how I learn and how I can change how I practice, which is super important...we are expected to do continuing professional development and take courses, but then it’s always read and learn around your cases. How am I supposed to learn around my cases when I can’t find out what the specialists thought of this unique situation that I can’t just open a textbook and read about.

##### Privacy, Confidentiality, and Security of the Record

The most common concern among participants regarded privacy and confidentiality. The extensive personal health information or personal information accessible in a central location, this being the record, increases the risk of a privacy breach:

I think about it, not only as a clinician, but as a user of healthcare as well.

Occupational therapist 1

According to registered nurse 1, with “a lot more information that is available to you as the healthcare provider, it would have to be ensured that only people who are part of the patient’s circle of care are accessing this information and that you are only accessing records that are applicable to the care that you are providing.” Nevertheless, most providers, including the nurses, physiotherapist, and occupational therapist, agreed that the benefits of the shared care record would outweigh the risks, and everyone could work together to make it as secure as possible.

##### Patient Portal Access

The concept of a patient portal, as described in the video shown to the participants, raised many questions and some hesitation among them. According to physician 1, “if the patients can see all of the notes that I write, that might change the way I document and I may be less comfortable putting the note in [to the record].” Understanding that patients will have access to their health data, questions revolved around the extent of access provided, whether to their entire chart and complete provider documentation or solely to scheduled appointments and their care plan:

...For instance, the other day I was printing a note for a patient knowing that her family member was going to be sitting with her reading it, she [the patient] asked me not to tell her family that she smokes weed...So, now I am changing my note, instead I copy and paste into a discharge note and then kept in my actual note all the facts so that I have that in her chart for other doctors to see because that’s important medical information.

Physician 1

Several end users expected that there would be system controls or protocols surrounding what information the patient could see or change themselves and what information the patient would not be able to change. Further discussions covered the documentation of sensitive patient information and how patients might respond to the presentation of such information:

...there might be certain things...in the record that maybe patients themselves would not want to see...if the patient was confused after a surgery and there was an episode of violence that was documented. It may almost be triggering or upsetting to them [the patients].

Registered nurse 2

Regardless, health care providers considered the inclusion of these types of documentation to be vital to the patient’s record:

I have a suspicion that this might be going on and I need to share that with my colleagues, because if they have a similar suspicion and it’s a pattern that’s important.

Physician 1

When considering patient access to records, participants emphasized the importance of patients being able to interpret the information correctly and objectively, especially regarding medical jargon. Participants suggested that clinicians may be inclined to use different terminology or a different writing style or reformulate the information in a meaningful way for patients:

...kind of helping socialize the clinicians to the new reality of the patients being able to read their notes more readily or easily. I think there would be value in having a discussion as a team about those types of changes.

Occupational therapist 1

## Discussion

### Overview

Concepts such as PHM, data security, and privacy can be complex to explain to individuals; however, they will become progressively essential to the design and delivery of health care. PHM is founded on interoperability, data sharing, and integration with diverse health sectors and services. Although people tend to understand the role and significance of EHRs, they may neglect the value of inputting accurate and high-quality data into them. PHM and primary health care strive for many of the same features, including person-centeredness; continuity; accessibility; and consideration of physical, mental, cultural, and social aspects of health, among others [2]. Health care providers commonly have a good understanding of the population that they serve, often living within the community themselves, and appreciate the needs and some of the determinants of health of these populations. A PHM approach rooted in quality data quantifies this understanding and enables an even deeper level of understanding [2,17]. As the vision for a shared care record using HIE technology starts coming to life, obtaining end users’ opinions and ideas will be imperative. End-user involvement in the record’s design; development; and, ultimately, operation will help simplify the adoption of changes and attain the goals of proactive and coordinated care that actively engages patients.

### Principal Findings

This study provides practical findings that will help emphasize factors that facilitate clinicians’ process of adaptation to the use of a shared care record. Considering the fast pace of health care, clinicians highly commended and admired a central location for real-time information availability that could promote efficiency through the effective use of time. The benefits of accessible retrieval of information were especially highlighted among end users practicing in the community setting. Discussions with end users brought forth the importance of an informed circle of care, promoting patient continuity of care, and more effective provision of care. Health care providers requested access to additional information that would help them in their practice, from medication lists and diagnostic imaging to social community and home care support, laboratory test results, and referrals. Discussions also brought forth questions regarding the interoperability of the record, its functional usability, and changes in workflows.

Adaptation to a shared care record was viewed positively by health care providers. Several end users spoke about the benefits of getting to know the new system through proper ongoing training using multifaceted approaches. Some of the approaches considered included videos, in-person and web-based computer sessions, and live user support options. The idea termed “super users” was brought forth, whereby colleagues who would be more acquainted with the software would function as support for their coworkers in the transition and adaptation to use of the record. End users wanted to understand the functionality of the record, the impact of changes on their daily workflows, and the consistency and accuracy of data across the record to maximize the utility and reliability of the clinical data. The main concerns of participants were the privacy, confidentiality, and security of the record and patient information interpretation through the patient portal.

A growing body of literature on the topic of patient access to health care provider electronic visit notes suggests that the active involvement of patients at the point of care can foster stronger patient-provider therapeutic partnerships. A study by Wolff et al [26] suggested that most patients reported benefits of reading provider notes, such as more agreement concerning treatment care plans, increased ability to formulate questions to ask their care providers, and more productive care discussions. Walker et al [27] brought forth challenges such as patients not being registered on portals to allow for access to notes or patients being unaware of provider notes being available to access. Nevertheless, the benefits of expanded patient access to clinical notes have been established, holding the potential to better support and involve patients in care, increase communication, and provide feelings of control and preparation for health care visits [26-28].

### Limitations

This study was limited in certain ways. The range of clinicians could have included various other providers within diverse health care settings to broaden the perspectives included. Furthermore, the application of voluntary response sampling in the recruitment of health care providers for this study is a limitation because of the possible sampling bias of respondents who volunteered, meaning that the study could have involved EHR advocates. Future research should involve a subsequent round of health care provider interviews once the record has a fully developed user interface design functioning across several systems involved with the HIE initial demonstration project. At this stage, health care provider interviews may offer further understanding of the functional usability of the shared care record once the providers can visualize and use it within the home database system they work in daily. These interviews could be geared toward comprehending how information design principles align with clinician workflows, patient information examinations, or decision-making in the medical environment. Building on this effort can help populations receive high-quality care while ensuring that it meets community needs.

### Conclusions

This study provided insights into health care providers’ perceptions of a shared care record and presented their reflections on the practical use and adaptation to the use of a shared care record. It is essential to bring end-user perspectives into the shared care record’s development, introduction, and maintenance, along with the training necessary to permit the use of the system. There is an urgent demand for high-quality, integrated, and timely health data allowing individuals, health care providers, and communities to be involved and informed partners in the provision and attainment of health care [17].

## Abbreviations

EHR: electronic health record
HIE: health information exchange
OHT: Ontario Health Team
PHM: population health management
